# 

**Published:** 1889-01-26

**Authors:** 


					r>
PERMANENTLY ENLARGED TO 32 PAGES.
ONE PENNY. . ^ ?"">
M0- A22, Vol. V.-
January 26th, 1889.
4 1L_JB Per Annum, 6s. 6d., post free. / /
Ditto per Ann. 7s. 6d., post free.

				

